# Cryo-electron tomography of stationary phase *Burkholderia thailandensis*

**DOI:** 10.17912/micropub.biology.001178

**Published:** 2024-04-24

**Authors:** Kanika Khanna, Matthew D. Welch

**Affiliations:** 1 Gladstone Institute of Virology, Gladstone Institutes, San Francisco, California, United States; 2 Department of Molecular and Cell Biology, University of California, Berkeley, Berkeley, California, United States

## Abstract

*Burkholderia* species belonging to the pseudomallei group include significant human and animal pathogens as well as the non-pathogenic species *Burkholderia thailandensis* . These bacteria co-opt the host cell machinery for their replication and spread between host cells. Thus, it is of interest to understand the structural features of these cells that contribute to host cell colonization and virulence. This study provides high-resolution cryo-electron tomograms of stationary phase *Burkholderia thailandensis* . It reveals the presence of compact nucleoids and storage granules, as well as examples of the type III secretion system and chemoreceptor arrays. The data can be used to investigate the near-atomic structure of stationary-phase bacterial macromolecules, such as ribosomes.

**Figure 1.**
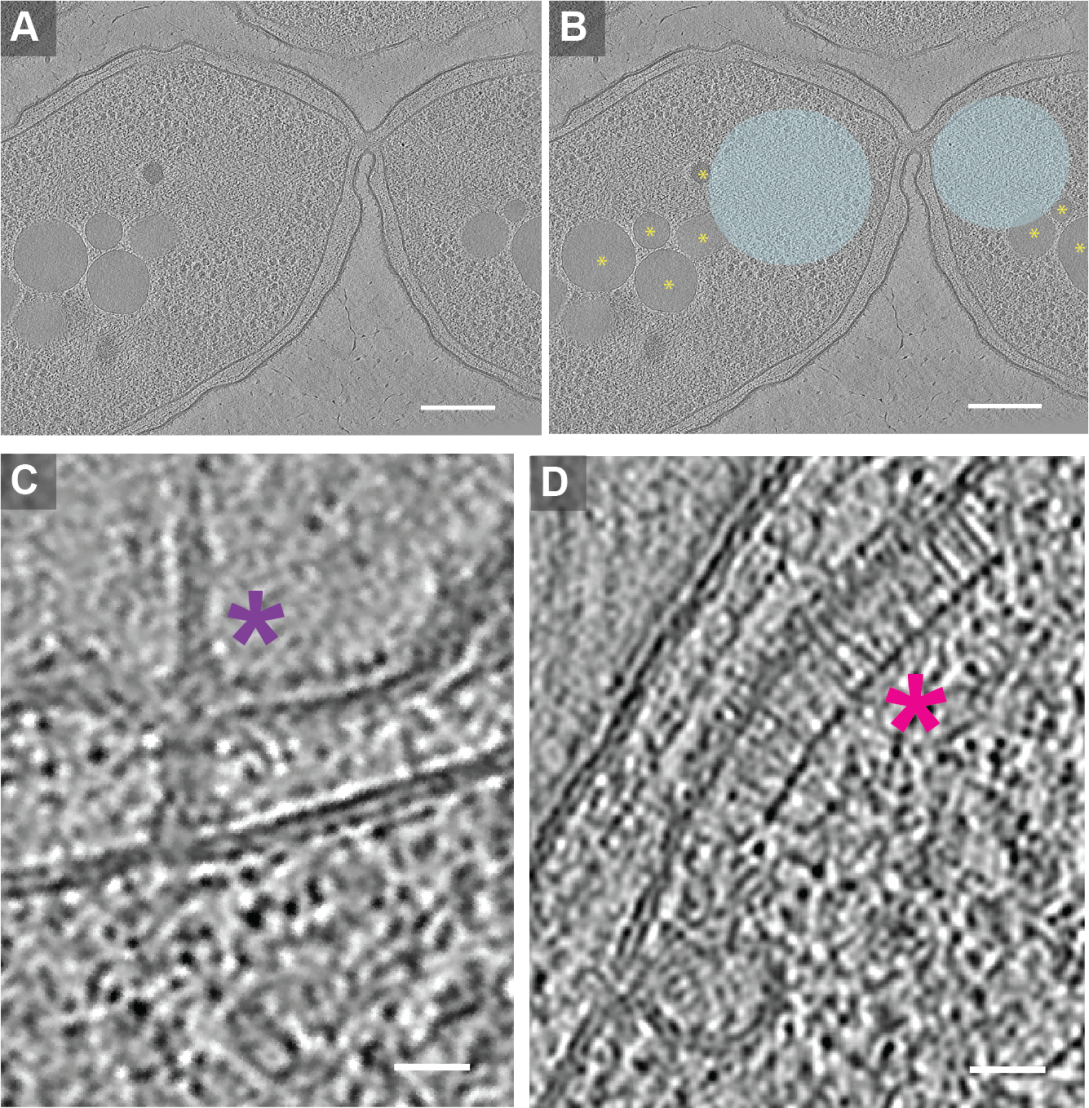
**
Figure 1. Cryo-electron tomograms of stationary phase *B. thailandensis* .** (A) Slice of a representative cryo-FIB milled tomogram of *B. thailandensis* . (B) Same as (A), annotated to depict storage granules (yellow asterisks) and condensed chromosomes segregating during cell division (blue). (C) Zoomed-in view of a *B. thailandensis* tomogram depicting a type III secretion system (purple asterisk). (D) Zoomed-in view of a *B. thailandensis* tomogram depicting chemoreceptor arrays (pink asterisk). Scale bars: (A,B): 200 nm (C,D): 25 nm.

## Description

*Burkholderia thailandensis* has been used as a model to advance our understanding of related pathogenic pseudomallei-group *Burkholderia* species. These include *Burkholderia pseudomallei* , which causes melioidosis, a potentially fatal disease prevalent in tropical regions, and *B. mallei* , which causes glanders in horses and other related animals (Galyov, Brett, and DeShazer 2010; Wiersinga et al. 2018; Khan et al. 2013) . Currently, there are no vaccines for disease prevention (Titball et al. 2017) , and infections caused by these bacteria are difficult to treat as they are resistant to many antibiotics (Rhodes and Schweizer 2016) . These Gram-negative facultative intracellular bacteria use virulence factors to hijack the host cellular machinery for replication and spread from cell to cell (Galyov, Brett, and DeShazer 2010; Willcocks et al. 2016) . While *B. thailandensis* encodes similar virulence factors (Haraga et al. 2008; Brett, DeShazer, and Woods 1998) , it is rarely pathogenic to humans (Glass et al. 2006) , making it a valuable model for studying the intracellular life cycle of pseudomallei-group *Burkholderia* in the laboratory.

Pseudomallei-group *Burkholderia* species are distinctive among bacterial pathogens in that they induce cell-cell fusion to spread between cells in tissues, forming multinucleate giant cells (MNGCs) (Kespichayawattana et al. 2000) . To do so, they require two activities. One is bacterial motility, either actin-based or flagellar (Benanti, Nguyen, and Welch 2015; Kespichayawattana et al. 2000; French et al. 2011; Toesca, French, and Miller 2014) . The other is the function of a type VI secretion system (T6SS), specifically T6SS-5 (Toesca, French, and Miller 2014; French et al. 2011; Kostow and Welch 2022; Schwarz et al. 2014) , a needle-like apparatus that secretes bacterial effector proteins from its tip (Jurėnas and Journet 2021). *B. thailandensis-induced* cell-cell fusion occurs within host cell plasma membrane protrusions (Kostow and Welch 2022) and requires T6SS-5 spike proteins VgrG5 (Schwarz et al. 2014; Toesca, French, and Miller 2014) and TagD5 (Kostow and Welch 2022) .

We set out to visualize *B. thailandensis* cell structures at a high resolution using cryo-electron tomography (cryo-ET) coupled with cryo-focused ion beam milling (cryo-FIB) (Khanna and Villa 2022; Keck, Enninga, and Swistak 2023) (see *Methods* ). We were particularly interested in visualizing the T6SS machinery, which was previously visualized by cryo-ET in *Vibrio cholerae* (Basler et al. 2012) and *Myxococcus xanthus* (Chang et al. 2017) and appears as cytoplasmic tubes resembling bacteriophage tails that are perpendicularly anchored in the bacterial membranes. To specifically visualize T6SS-5, we used *B. thailandensis* strain E264 ΔT6SS-1,2,4,6, which was engineered with inactivating mutations in T6SS-1, 2, 4, and 6, leaving only T6SS-5 active (Schwarz et al. 2010) . The expression of *B. thailandensis* T6SS-5 is tightly controlled and induced exclusively inside host cells (Chen et al. 2011; Lennings, West, and Schwarz 2018; Schwarz et al. 2014) . VirA, the principal regulator of T6SS-5 expression, senses the presence of reduced glutathione (GSH) in the host cytosol, initiating the expression of T6SS-5 genes (Wong, Chen, and Gan 2015) . This knowledge can be leveraged to express T6SS-5 genes in *B. thailandensis* grown in broth culture without host cells by supplementing the media with GSH (Wong, Chen, and Gan 2015; Kostow and Welch 2022) . Hence, we decided to use cryo-FIB-ET to visualize *B. thailandensis* grown in broth with GSH to increase the chances of visualizing T6SS-5.

We acquired 13 high-quality tomograms of *B. thailandensis* . All of our tomograms displayed characteristics typical of stationary-phase bacteria ( Figure 1 ). This includes the presence of condensed DNA (Bauda et al. 2024) , which is suggested to serve as a strategy to safeguard DNA from potential damage (Lee et al. 2015; Almiron et al. 1992; Janissen et al. 2018) . It also includes the presence of other spherical structures, potentially either polyphosphate (polyP) or polyhydroxyalkanoate (PHA) granules (Racki et al. 2017; Chawla et al. 2023; Achbergerová and Nahálka 2011; Tocheva et al. 2013). These presumed PolyP/PHA granules exhibited irregular shapes and heterogeneity in size, ranging from approximately 50 to 400 nm. The granules also varied in number and were located non-uniformly within the cell. Other observed structures include numerous ribosomes, chromosome segregation during the final stages of cell division ( Figure 1A,B ), an instance of the type III secretion system (T3SS) ( Figure 1C ), and an instance of chemoreceptor arrays ( Figure 1D ).

Regrettably, we did not visualize T6SS-5 in our dataset. Several possibilities may explain our inability to visualize T6SS-5. Firstly, the limited sample size and the thinning of cells (to ~200 nm) during cryo-FIB milling, allowing us to capture only ~20% of the cell, could have contributed to the inability to observe individual examples of T6SS-5. Increased data acquisition might address this limitation. Consistent with this notion, a prior study detected the T6SS in whole *M. xanthus* cells in only 53 out of 1650 tomograms (~3.2%), suggesting a low probability of detecting T6SS in cells in general (Chang et al. 2017) . In the future, we anticipate that using correlative light microscopy to identify cells expressing T6SS-5 in *B. thailandensis* can help alleviate this issue. Secondly, while the expression of VgrG-5 was observed under our growth conditions by western blotting (Kostow and Welch 2022) , this might not necessarily correspond to the assembly of the complete T6SS-5 apparatus, which may depend on factors beyond the addition of GSH to the growth media. Lastly, it is plausible that optimizing our growth conditions to ensure bacterial cells are not in the stationary phase when GSH is introduced to the media could enhance our ability to visualize T6SS-5.

Nevertheless, we anticipate that these tomograms will be a valuable resource for the scientific community interested in studying bacterial structures for cells in stationary phase, for example, in deciphering the structure of stationary-phase ribosomes. We have deposited binned tomograms in the Electron Microscopy Data Bank (EMDB) with accession code EMD-44416 and aligned tilt series in the Electron Microscopy Public Image Archive (EMPIAR) database with accession code EMPIAR-11957.

## Methods

*Bacterial cell culture*

*B. thailandensis* E264 ΔT6SS-1,2,4,6 strain was generously provided by the lab of Joseph D. Mougous (Schwarz et al. 2010) . For expression of VgrG5 in broth, the same procedure was followed as was previously reported (Kostow and Welch 2022) . Briefly, *B. thailandensis* was grown overnight in LB broth, followed by 1:10 dilution in the morning. After 2 h, cultures were split into two tubes with 2 mM reduced L-glutathione (GSH, Sigma-Aldrich, G4251) added in one of them. Cultures were further grown for 4 h for cryo-ET, as described below.

*Tomography Sample Preparation and Data Acquisition*

Holey carbon-coated QUANTIFOIL® R 2/1 copper grids (Electron Microscopy Sciences, Q350CR1) were glow-discharged using Pelco easiGlow ^TM^ Glow Discharge Cleaning System, and 6 µl of GSH-induced *B. thailandensis* culture was deposited on each grid. The sample was blotted from the side of the grid opposite to the cells using Whatman No. 1 filter paper to remove excess liquid such that cells form a monolayer on the grid. Vitrification was done in liquid ethane using a manual freeze-plunger at the Donner Cryo-EM Facility, Lawrence Berkeley National Laboratory (LBNL). The samples were stored in liquid nitrogen until further use.

Subsequently, for cryo-FIB milling, vitrified cells were transported to the Stanford-SLAC CryoET Specimen Preparation Center and milled using Aquilos 2 cryo-FIB (ThermoFisher Scientific) manually, as described in previous workflows (Wagner et al. 2020; Khanna et al. 2021) . Briefly, grids were sputter-coated with metallic platinum, followed by coating with a ~500 nm organometallic platinum layer using a gas injection system and another round of sputter-coating with metallic platinum. Lamellae with thickness ranging from ~150-200 nm were prepared using a 30 kV focused ion beam, gradually reducing the beam current from 500 pA (rough milling) to 10 pA (polishing). Micro-expansion joints were also milled to prevent lamellae bending and fracture (Wolff et al. 2019) .

Milled samples were transported to UW-Madison Cryo-Electron Microscopy Research Center (CEMRC) for cryo-ET data acquisition using 300 kV Titan Krios transmission electron microscope (ThermoFisher Scientific) equipped with a K3 camera and an energy filter (both from Gatan). A slit width of 20 eV was used for data acquisition in 0.5 binning mode on the K3 detector. SerialEM 4.1 software was used for data acquisition (Mastronarde 2005) . Tilt series were acquired at 3.726 Å pixel size using a dose-symmetric (Hagen, Wan, and Briggs 2017) scheme with 3° increments starting from 0° relative to the lamella pretilt, with the total dose applied to the sample ranging from 70-80 e-/Å² and a defocus of -5 µm. MotionCor2 was used to correct individual tilt frames (Zheng et al. 2017) and AreTomo for tilt-series alignment and reconstruction with dose weighting (Zheng et al. 2022) . Final reconstruction and visualization was done using the patch-tracking method in IMOD 4.11 (Kremer, Mastronarde, and McIntosh 1996) .
